# The grammar of the gallery: room corners influence the mental representation of picture arrangements

**DOI:** 10.3389/fpsyg.2026.1728757

**Published:** 2026-02-11

**Authors:** Bärbel Garsoffky, Marco Benkert, Stephan Schwan

**Affiliations:** Realistic Depictions Lab, Leibniz-Institut für Wissensmedien, Tübingen, Germany

**Keywords:** architecture, memory, room design, viewing behavior, virtual reality

## Abstract

Especially informal learning settings, such as exhibitions, (virtual) museums or historical places, arrange interesting visual content not only in front of but also all around the visitor. Visitors move through these three-dimensional places and determine in which order and how long they pay attention to the different pieces of information. In this article we ask whether and how architectural characteristics influence the cognitive processing of visitors when they deal with the presented content. In the first part of the article, several experiments describe the detrimental effect of corners on memory for related pieces of information; pairs of information were memorized better if they were presented within one wall instead of on two adjacent walls with a corner between them. Further, the findings of previous studies are summarized, suggesting that this corner effect could be overcome by restricting learners in where they could orient their central field of view or by grouping the informational units across corners using spatial gaps on the walls. In the second part of the article, a study is presented in more detail which tested whether the corner effect could also be overcome by thoughtful lighting of the rooms. Pairs of pictures were arranged on the walls of a virtual room, and learners had to memorize where the two pictures forming a pair were placed. Lighting was varied, and it was expected that in evenly lit rooms the pairs of pictures would be remembered better if arranged within one wall instead of across two adjacent walls, whereas in rooms with brighter light in the corners this effect should be weaker. The findings replicated the corner effect on memory, but showed no influence of lighting.

## 1 Introduction

Architectural space is static, whereas events are dynamic. How can architectural characteristics constitute dynamic events? Space is often assumed to be a kind of three-dimensional container in which events take place. But architectures are not simply neutral containers. Instead, they are places that have certain characteristics and structures which may influence behavioral patterns and resulting mental representations. As we move or walk through buildings, architecture sequentially unfolds and structures our experience, creating discernible events, as the seminal study of walking through doorways and event cognition by Radvansky and Copeland (2006) convincingly demonstrated. In two experiments participants walked through a virtual environment and actively picked up and carried objects from one room to other rooms, thereby passing doorways. Findings showed, that participants remembered an object better, if they were actually carrying this object than when they had already parked it, and that this was especially the case if they had just passed through a doorway. That is, it seems that participants updated their situational model, i.e., the mental representation of the objects and entities and their relations in an event, when entering a new room, thereby again becoming aware of the actual object they were carrying at that point of time. Radvansky and Copeland (2006) conclude that participants develop a situational model of the environment they are interacting with, and that a spatial change makes people to update their situational model. This updating requires cognitive resources and occurred even if the task did not emphasize to update the spatial environment (Radvansky and Copeland, 2006). It seems plausible to examine if this observation holds not only for doorways but also for other architectural features such as turns of aisles, stairways, or, as the most simple cases, even room corners.

A prototypical architectural setup is an art exhibition gallery. At first sight, the layout is simple: an empty room with four white walls – a “white cube” (O’Doherty, 1999) – with paintings and other artworks hanging on the walls. Yet, curatorial practice tells us that the arrangement of the artworks is a complex craft that requires a considerable level of expertise. The arrangement of paintings on the walls clearly influences visitors’ behavior in terms of their movements and stops, as evidenced by timing and tracking studies (Yalowitz and Bronnenkant, 2009). In addition, inspecting works of art in a gallery one-by-one does not actually require bodily movement, but can also be realized by moving one’s head and eyes from a stationary position (Linden and Wagemans, 2024).

In contrast to settings where information is presented directly in front of a person (think of lectures, books or face-to-face conversations), in museums, historical places or white cube galleries people are surrounded by information. This is also the case with virtual and mixed reality settings, which convey the impression of being immersed in three-dimensional space filled with information displays. Accordingly, the extended model of cognitive load from Choi et al. (2014) postulates that not only the specific task and the learner but also the surrounding space in a learning situation influences cognitive load and can therefore support or hinder effective information processing.

Combined with physiological data, timing and tracking studies demonstrate the impact of these behavioral patterns on arousal and emotion (Tschacher et al., 2012). Findings also indicate that characteristics of the spatial layout play an important role in cognition. For example, McNamara et al. (1989) found that the spatial memory of complex arrays of objects arranged in sub-areas encompasses a hierarchical component, i.e., objects belonging to one sub-area tend to be presented in one subtree, thereby influencing distance estimations and priming. An example of an architectural feature which influences cognitive processes is ceiling height. Meyers-Levy and Zhu (2007) showed that a higher ceiling in a room primed concepts of freedom, thereby prompting participants to process presented contents more relationally whereas a lower ceiling height primed concepts of confinement and prompted participants to more item-specific processing of contents.

## 2 Memory of related information in 3-dimensional rooms

### 2.1 The “corner effect”

In a gallery of the “white cube” type, one of the most basic features is the separation of adjacent walls by room corners. Based on the assumption that room corners constitute salient architectural borders which interrupt the continuum of displayed information (for example, sequences of artworks), we conducted a series of studies (Garsoffky and Schwan, 2024) which aimed to investigate the influence of room corners on the mental representation of information displayed on the walls. As an experimental paradigm we chose a variant of the widespread “memory” game for children, where players try to memorize which hidden cards on a table show the same motif. In the experiments of Garsoffky and Schwan (2024), on the walls of a room, several pairs of pictures of everyday objects were displayed, i.e., there were always two cards showing the same motif, e.g., the same photo of an apple, forming a pair. These picture pairs were arranged on three walls in two rows with six pictures in each row, and the two cards forming a pair were arranged either within one wall or across two adjacent walls, i.e., with a corner in between them. Further, the two pictures which form a pair are never placed next to each other, but were always separated by other pictures between the two. Participants knew from a training block that, after learning the arrangement of pictures in a room, their memory of the pairs would be tested and they had to remember where on the walls the pairs of pictures were presented. This procedure was repeated eight times (eight rooms) with different sets of picture pairs (depicting e.g., animals, sweets, and fruits). In every room there was the same procedure. During the learning phase participants had to look around and memorize the location of the picture pairs on the walls of this room. In the following test phase, all cards were turned so only their black back was visible. Then in every test trial, all the cards on the walls only showed their black backs, but one card showed its motif (i.e., “was turned around”) and another card (still only black back visible) was marked by an orange frame. The participants then had to decide if the two cards (when the motif of the marked card would also become visible) would show the same motif (“yes”) or not (“no”). So, to perform the yes/no task, participants had to remember, where on the walls the two cards forming a pair were located.

The three studies of Garsoffky and Schwan (2024) were conducted as online experiments, in which participants explored a three-dimensional virtual room on a flat computer screen. In experiments 1 and 2, during the learning phase of each room, participants could freely turn 360 degrees in the room by pressing the arrow keys. Results showed that picture pairs were better memorized if both pictures of a pair were arranged on one wall instead of being arranged on two adjacent walls with a corner between them. The descriptive data indicated that during learning the participants preferred to orient their central field of view toward the middle of the walls, rather than toward the room corners, i.e., that they seemed to process the stimuli in groups “wall by wall.” Therefore, the third online experiment limited where participants could orient their field of view during learning (Garsoffky and Schwan, 2024); they could learn the pictures either in a wall-by-wall manner (by turning their central field of view from one wall to the next) or in a “corner-wise” manner (by turning their central field of view from one corner to the next). The amount of information which a participant saw in his or her field of view was always the same, namely two rows of pictures with six pictures per row. In this experiment the detrimental effect of corners on remembering the pairs was also found in the scenario where participants learned the pairs in the rooms wall by wall. But when they were forced to memorize the content by skipping their field of view from corner to corner, the detrimental effect of corner vanished.

Taken together, these three experiments (Garsoffky and Schwan, 2024) indicated a corner effect: the simple fact that there was a corner between related information (in this case, the two pictures forming a pair) can influence people’s memory of this relation. Hence, this basic architectural feature had a clear impact on the memory of the related content presented on the walls of the rooms, even though the participants knew beforehand that pairs could be arranged both on a single wall or across two walls. The results of the third study further indicate that the corner effect is mainly based on behavioral patterns: when participants could freely explore the room, they tended to process the pictures and their spatial position in a wall-by-wall manner, with corners constituting borders which separated information processing events. But if they were forced to orient their central field of view toward the corners (as in experiment 3, Garsoffky and Schwan, 2024), the corner effect vanished. This suggests that the corners of the rooms seem to influence exploration behavior and this in turn is mirrored in memory processes.

### 2.2 Corner effects and spacing in virtual reality

The goal of another study (Garsoffky et al., 2025) was twofold. The first question posed was whether the findings found in three-dimensional virtual environments and experienced on two-dimensional screens (Garsoffky and Schwan, 2024) could be replicated in immersive virtual three-dimensional rooms, when participants wore VR-glasses and explored the arrangement of information around them by turning their bodies 360 degrees instead of skipping with the arrow keys. This situation was more similar to the experience of navigating rooms in everyday life; there was nothing else in the field of view other than the room with its pictorial information, and the viewer looked around by turning his or her head and body instead of interacting with arrow keys.

The second question of this previous study (Garsoffky et al., 2025) was to investigate the detrimental effect of corners on memory more closely. The results of Garsoffky and Schwan (2024) indicated that participants tended to orient their central field of view more frequently toward the middle of walls then toward corners. In other words, participants seemed to build subgroups of pictures when memorizing the stimuli and these sub-groups were defined by walls. This effect disappeared in experiment 3 (Garsoffky and Schwan, 2024) in which participants were forced to orient their central field of view toward the corners instead. Based on this finding, it was reasoned that the detrimental effect of corners should also vanish if the spatial distances between stimuli supported grouping the stimuli in subgroups across corners instead of through a wall-by-wall grouping. Gestalt psychology, and in particular Wertheimer (1938), formulated rules regarding how visually dispersed stimuli are perceived and grouped together, and postulated that proximity is one important factor that influences which stimuli are perceived as belonging to one group. Palmer (1992) formulated the principle of common region and postulated, that stimuli which are perceived as belonging to the same spatial region are processed as belonging together. The findings in Garsoffky and Schwan (2024) similarly suggested that stimuli arranged on the same wall also tended to be perceived as belonging to the same group, whereas pairs of pictures arranged across two walls were perceived as belonging to different subgroups. Therefore, other types of grouping stimuli should also influence the impact of the arrangement of pictures on walls on memory.

**FIGURE 1:**
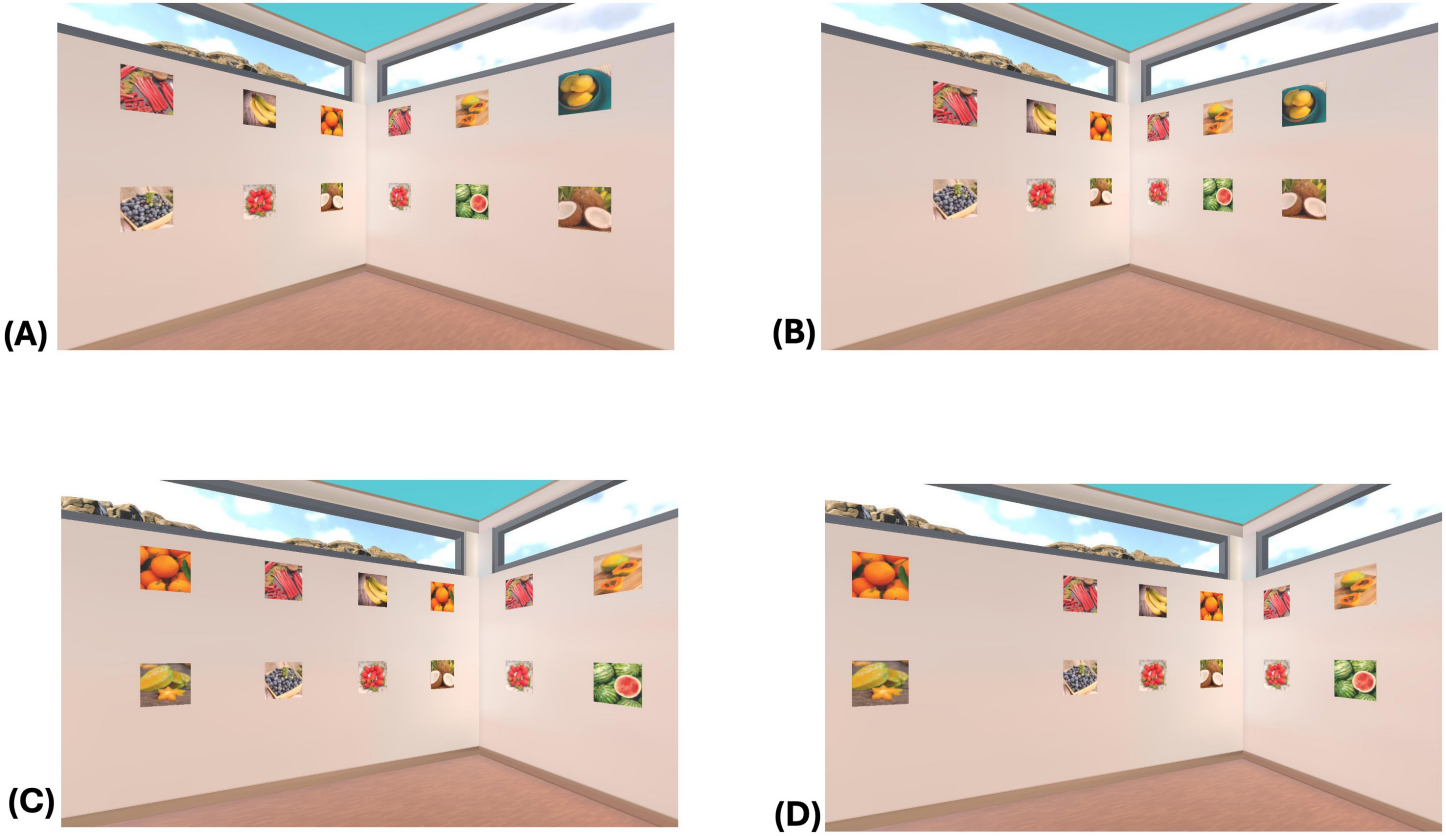
Picture arrangements in the experimental setting in the experiment on grouping (Garsoffky et al., 2025). **(A,C)** Even arrangement, **(B,D)** uneven arrangement. **(A,B)** Pictures of a pair across walls (motif rhubarb), **(C,D)** pictures of a pair within one wall (motif tangerines). Copyright EDULAERN2025.

In a fully immersive virtual reality setting with participants wearing Quest 3 glasses (Garsoffky et al., 2025), pictures forming a pair were again either arranged on one or across two walls. In addition, it was varied whether the twelve pictures on one wall were spaced evenly or unevenly, i.e., whether there was a greater distance between the pictures in the middle of the wall or not. In half of the learning rooms only the corners structured the stimuli, while in the other half of the rooms the greater distance between stimuli in the middle of each wall implied an alternative grouping. The distance between the two pictures forming a pair was kept constant; there were always two other pictures between the two pictures that formed a pair [see Figure 1 presenting the stimulus material used by Garsoffky et al. (2025)].

The findings (Garsoffky et al., 2025) showed that memory of the picture pairs was influenced by both the arrangement of the picture pairs on one or across two walls and the spacing between the pictures on the walls: The significant interaction between these two factors revealed that, if all pictures were arranged evenly on the walls, the pairs of pictures were memorized better if they were arranged on one instead of across two walls. This result replicated the finding concerning the detrimental effect of corners on memory for pairs found by Garsoffky and Schwan (2024). However, the detrimental effect of corners vanished if the pictures were additionally arranged in groups on each wall; if the pictures were unevenly spaced alongside each other, memory did not differ significantly between picture pairs on one or across two walls. Furthermore, for picture pairs arranged on one wall, this uneven spacing led to poorer memory of the pairs compared to the arrangement of pictures which were equally spaced. This result is plausible because, whenever pictures were unevenly spaced on the walls, the two pictures forming a pair within one wall were separated by a larger spatial gap, i.e., they belonged to two different visual groups. In contrast, if the picture pairs were presented on one wall and there was an even picture arrangement, all the pictures on the wall were placed at the same spatial distance between them and therefore there was no visual grouping of the pictures arranged on one wall.

**FIGURE 2:**
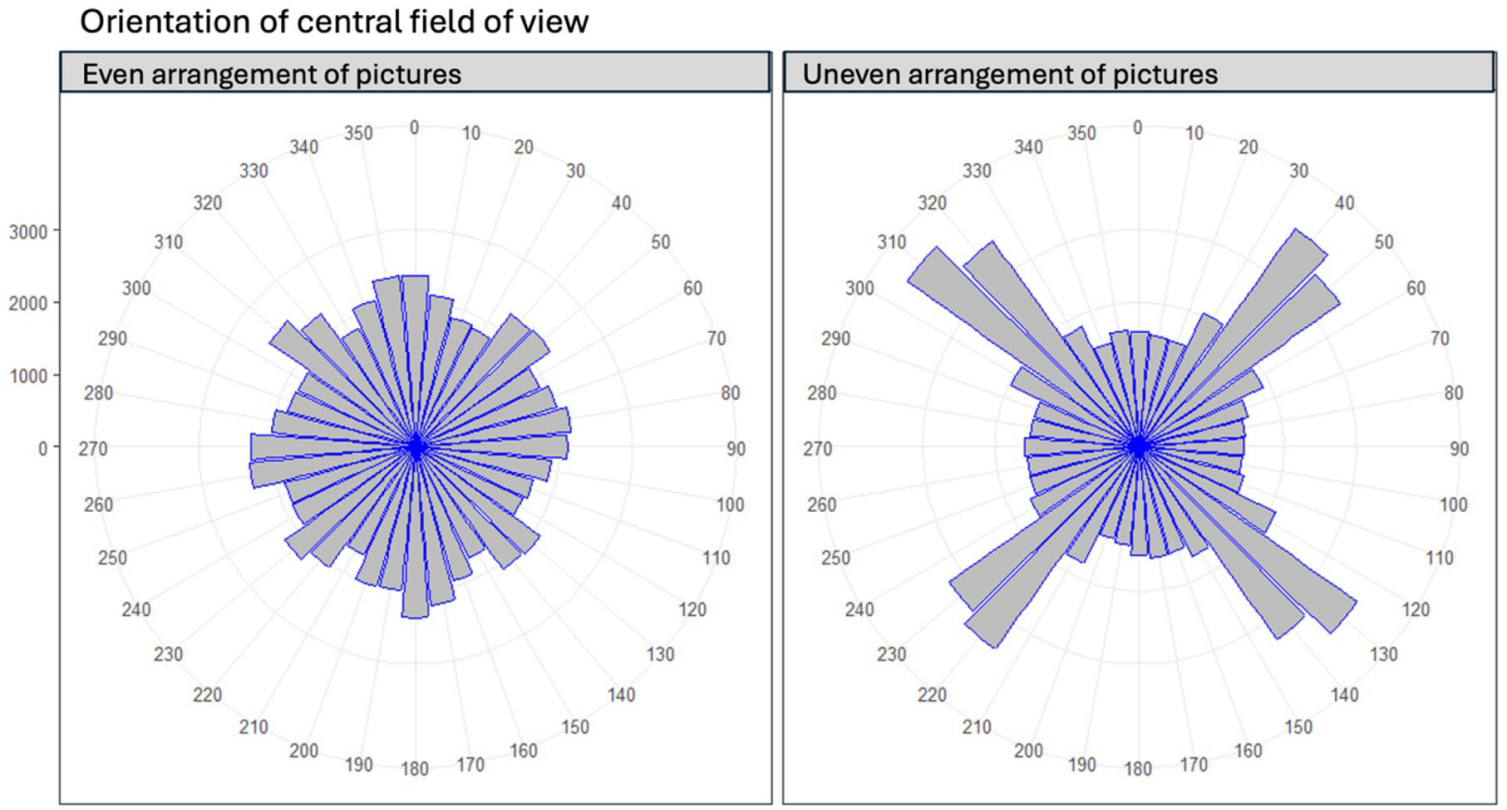
Descriptive data of orientation of central field of view in the experiment on grouping (Garsoffky et al., 2025). Number of views for every participant every half second. 0°, 90°, 180° and 270° indicating middle of the four walls, 45°, 135°, 225° and 315° indicating straight toward a corner.

Importantly, the descriptive data of where the participants oriented their central field of view during learning (Garsoffky et al., 2025) indicated that the larger distances between pictures in the middle of the walls encouraged participants to look toward the corners more often, resulting in the situation that pictures to the left and right of a corner were more often in the same field of view at the same time and therefore might have been perceived as a group [see Figure 2 presenting descriptive data of Garsoffky et al. (2025)]. Again, these effects seemed to be based on behavioral patterns; the different arrangements of pictures (even vs. uneven spacing) led to different viewing behaviors. In the evenly spaced condition, participants again tended to process the pictures and their spatial position in a wall-by-wall manner, with corners constituting borders between separate, wall-based information processing events. In contrast, the uneven spacing of pictures on the walls made viewers orient their field of view more often toward the corners, thereby reducing the corner effect. In other words, it seems that the corner effect could be counteracted by thoughtful grouping of the stimuli on the walls (Garsoffky et al., 2025). But, in some informal learning settings it may be impossible to simply re-arrange informational content in this way. The question remains in these cases: might the corner effect also be influenced by guiding the viewers’ visual attention during learning?

## 3 Guiding visual attention by lighting design

In the experiment reported here, the goal was to investigate whether the detrimental effect of corners on memory could also be overcome by guiding the viewers’ visual attention through careful lighting design in the learning phase. Furthermore, it was examined whether the corner effect on memory could again be replicated in immersive three-dimensional environments, i.e., when participants were positioned in the middle of a room surrounded by visual information. Therefore, the participants came into the lab and experienced the (virtual) rooms wearing high immersive VR-glasses (Quest 1).

Based on the metaphor of the attentional beam (Eriksen and Yeh, 1985; Posner, 1980), the idea behind this study was to use lighting to guide where participants oriented their field of view in a virtual room and how they processed information arranged on the walls. Examining the lighting effects is especially important for virtual rooms, because lighting offers several possibilities to design virtual worlds more realistic and immersive. It was expected that, if the virtual rooms were evenly lit, participants would memorize the picture pairs better if they were arranged on one wall instead of across two walls because they would presumably orient their field of view toward the middle of the walls [findings of Garsoffky and Schwan (2024)]. In contrast, it was assumed that a spotlight could guide the orientation of the visual field. It was hypothesized that, if the corners were lit more brightly than the middle of the walls, participants would memorize picture pairs arranged across two walls better than picture pairs that were arranged on one wall, or at least that there would no longer be an advantage in the memory of picture pairs on one wall compared to pairs across two walls, because they would orient their visual attention more often toward the corners.

### 3.1 Participants

The study was supported by the institute’s local ethics committee, #LEK 2023/044, and was preregistered before data collection on AsPredicted, #152271. The number of participants was calculated using G-Power 3.1 (Faul et al., 2007; Rasch et al., 2021). This calculation was based on the partial eta square = 0.126 for the interaction effect of wall arrangement × viewing direction from the third experiment reported in Garsoffky and Schwan (2024), which showed that the corner effect only appeared if participants were forced to look straight toward the middle of the walls, but disappeared if participants were forced to look straight into the corners. The planned experiment assumes that lighting guided the orientation of the field of view analogously to the forced viewing direction in experiment 3. According to Rasch et al. (2021), the number of participants for an expected interaction of two within factors can be calculated with G-Power by correcting the *f*-value accordingly. Alpha was set to 0.05 and 1−beta to 0.95. This resulted in a calculated sample size of *N* = 48.

Participants were recruited via an online platform for study participation. Forty-eight participants took part in single sessions at the institute’s lab, each lasting approximately 1 h. The average age was 25.5 years (minimum = 18 years, maximum = 66 years), while 16 of the participants were male and 32 female.

Data can be found on OSF^1^.

### 3.2 Material and procedure

**FIGURE 3:**
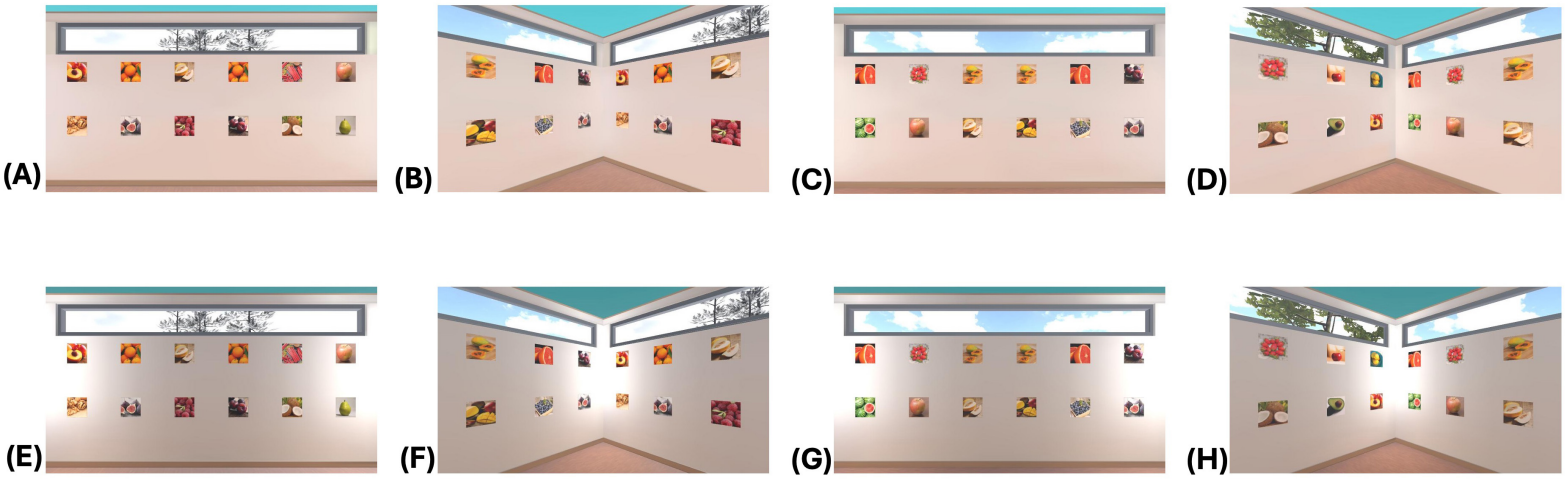
The stimulus material used in the experiment on visual attention presented in this paper. **(A–D)** Room lit evenly; **(E–H)** corner light. **(A,E)** Within wall and 1 between (tangerines); **(B,F)** across wall and 1 between (fig fruit); **(C,G)** within wall and 3 between (orange); **(D,H)** across wall and 3 between (strawberries).

The materials, procedure and analyses were similar to the experiments in Garsoffky and Schwan (2024) and the experiment in Garsoffky et al. (2025). In virtual rooms, which were programmed with Unity, several picture pairs of everyday objects (THINGS database, Hebart et al., 2019) were arranged on the four walls, and the rooms were lit either evenly or with a brighter beam in the corners (Figure 3). After a training phase, each participant saw six virtual rooms one after the other. In each room there were 48 pictures displayed according to one category of everyday objects (e.g., sweets, vegetable, animals). Each room looked exactly the same, except for the pictures on the walls. At the top of the walls there were (virtual) windows through which different natural environments were visible, e.g., a sky and trees to help participants orient themselves in the (doorless) square rooms. The pictures were arranged in two rows with six pictures on each wall. Each picture appeared twice, for example the same photo of an apple, forming a pair. In each room 48 pictures were displayed, or 24 pairs.

The same number of two pictures forming a pair was placed on one wall or across two adjacent walls, i.e., with a corner between the two pictures (factor arrangement on one wall/across two walls). Further, between the two pictures of a pair there was either one or three other pictures (factor distance 1/3 pictures between). Half of the rooms were evenly lit, while the other rooms were lit more brightly in the corners than in the middle of the walls (factor lighting evenly/corner light). This resulted in a 2 × 2 × 2 within-Design. Each participant either first saw three evenly lit rooms and then three rooms with corner lighting, or the first three rooms with corner lighting and then three rooms that were evenly lit. For each participant it was randomized in which lighting condition he or she saw which picture topic (e.g., sweets, animals, vegetables) as well as where in the room the specific picture pairs were placed that were later tested. For example, the pair of apples was arranged for some participants on one wall and for other participants across two walls.

At the beginning of an experimental session, after obtaining informed consent, each participant was placed on a freely rotating office chair and put on the Head Mounted Display Meta Quest 1 Headset (HMD) to see the virtual reality. The presentation in the HMD started with two experimental rooms, in which all conditions were pre-set and the participants could get used to the task. The experimental procedure was the same in every room; the participant saw the virtual room in the HMD while being in the middle of the room. Then the learning phase began; for 2 min and 40 s, the participant could freely turn around 360 degrees and memorize the location of the pairs in the room. This was followed by the test phase, in which all the pictures were turned so that only their black backs were visible and memory of the pairs was measured. Twelve test trials came next (8 experimental trials and 4 filler trials). For each trial there was an orange beam presented on the floor, so the participant knew in which direction he or she had to look. Then one picture became visible (e.g., the apple) and another picture (which had only its black back visible) was highlighted by an orange frame. The participant then had to indicate whether or not the two pictures would form a pair if both pictures would be visible, by pressing the left or the right handheld controller key. Half of the two cards forming the test trials were arranged on one wall, the other half across two walls; half of them were separated by one other picture between them, and the other half by 3 more pictures between them. Further, half of the two pictures in the test trails formed a pair (right answer “yes”) and half of them did not (right answer “no”). The four filler trials presented two cards in different rows to prevent participants from memorizing the pictures in a purely row-based manner. The filler items were excluded before analyzing the data.

Further, additionally to the memory activity, every half second it was measured where in the room each participant oriented his or her central field of view during the learning phase.

### 3.3 Results and discussion

To measure the memory of pairs, sensitivity A’ was calculated. This measure is based on signal detection theory (Macmillan and Creelman, 2005; Stanislaw and Todorov, 1999) and considers how well a participant can differentiate between “pairs” and “no-pairs,” taking into account not only “hits” (i.e., “yes” answers for pairs) but also “false alarms” (i.e., “yes” answers for no-pairs). Because of the relative low number of measurements a non-parametrical measure was used and sensitivity was calculated according to the following formula (Stanislaw and Todorov, 1999, p. 142):

When H ≥ F


$$A^{\prime }=0.5+\left(\left(H-F\right)\,\left(1+H-F\right)\right)/4\,H\,\left(1-F\right)$$

When H < F


$$A^{\prime }=0.5-\left(\left(F-H\right)\,\left(1+F-H\right)\right)/4\,F\,\left(1-H\right)$$

With *H* = hit rate, found by dividing the number of Hits by the total number of signal trials, and *F* = false-alarm rate, found by dividing the number of false alarms by the total number of noise trials (Stanislaw and Todorov, 1999).

**FIGURE 4:**
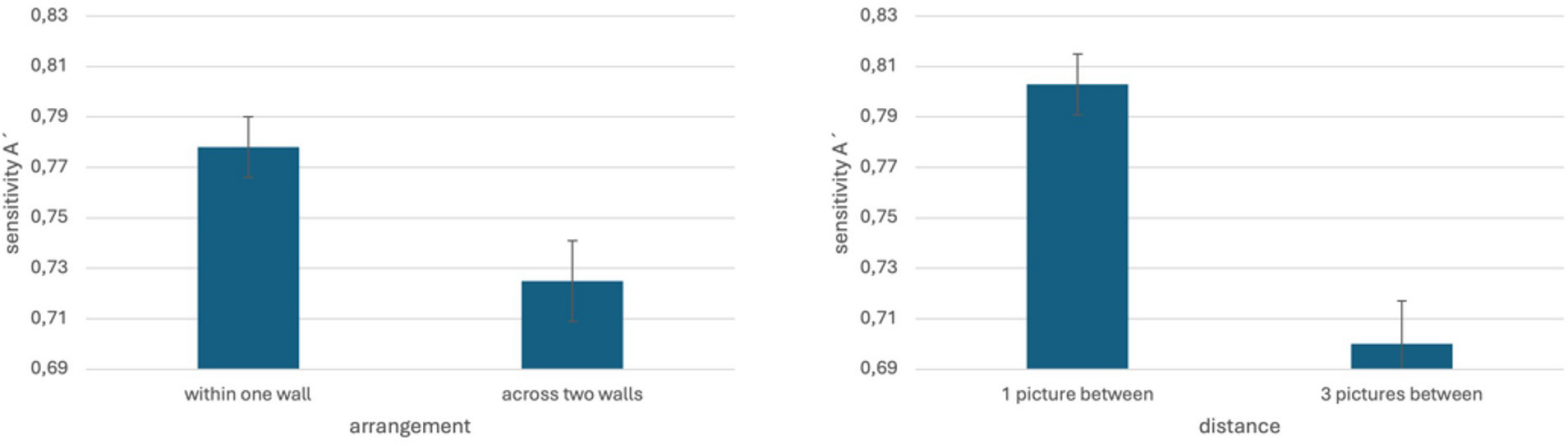
Significant main effects of arrangement and distance on sensitivity in the experiment on visual attention presented in this paper: pairs of pictures were remembered better, if they were arranged within one instead of across two walls, and if there was only one more other picture between them instead of three other pictures. Error bars indicating standard errors.

First, for every participant his or her overall sensitivity A’ was calculated. One participant had an overall sensitivity A’ < 0.5. Because this can be a hint that the participant confused the response buttons, the data of this participant were excluded from analysis. Then a repeated measures analysis of variance (ANOVA) with the factors “wall arrangement” (on one wall vs. across two adjacent walls), “distance” (one vs. three other pictures between them) and “lighting” (evenly lit vs. corner lighting) was calculated. There were two significant effects (Figure 4): the main effect of “wall arrangement” [F(1, 46) = 12.242, MSE = 0.021, *p* = 0.001, η_*p*_^2^ = 0.21] showed higher sensitivity if the two cards were presented on the same wall (*M* = 0.778, SE = 0.012) than if the two cards were presented on two adjacent walls (*M* = 0.725, SE = 0.016). The main effect of “distance” [F(1, 46) = 36.615, MSE = 0.027, *p* < 0.001, η_*p*_^2^ = 0.443] showed higher sensitivity if there was only one more picture between the cards (*M* = 0.803, SE = 0.012) than if there were three more pictures between the cards (*M* = 0.7, SE = 0.017). There were no other significant main effects or interactions in this analysis (see Table 1).

**TABLE 1:** Repeated measures analysis of variance (ANOVA) results.

Source	df	dfe	MSE	F	*P*	η _*p*_^2^
Lighting (L)	1	46	0.017	3.279	0.077	0.067
Wall arrangement (WA)	1	46	0.021	12.242*	0.001	0.210
Distance (D)	1	46	0.027	36.615*	<0.001	0.443
L × WA	1	46	0.020	0.007	0.935	0.000
L × D	1	46	0.017	0.879	0.353	0.019
WA × D	1	46	0.018	0.222	0.640	0.005
L × WA × D	1	46	0.018	1.084	0.303	0.023

*p < 0.05; MSE = mean square error; η_*p*_^2^ = partial eta squared; dfe = df error.

**FIGURE 5:**
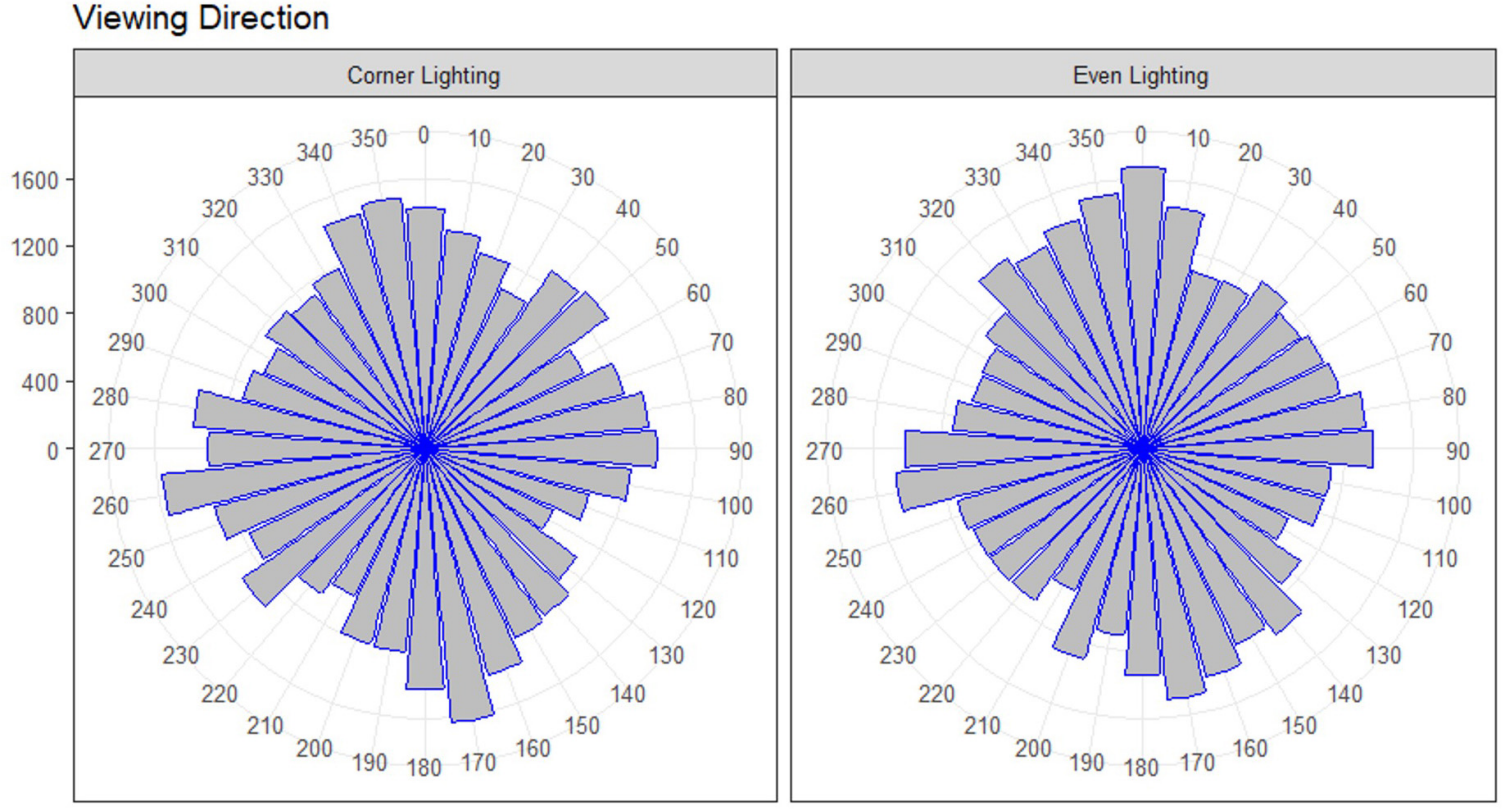
Lighting does not seem to influence the orientation of the central field of view during learning in the experiment on visual attention presented in this paper: number of views for every participant every half second. 0°, 90°, 180° and 270° indicating middle of the four walls, 45°, 135°, 225° and 315° indicating straight toward a corner.

Further, it was examined descriptively where participants oriented their central field of view during learning. The first 5 s in each room were not included in the descriptive analysis, because every participant started a new room with a random orientation and it was expected that participants used the first few seconds to orient their central field of view to a desirable viewpoint. Looking exploratively at these data, lighting did not seem to influence the orientation of the central field of view (Figure 5).

Our findings replicated the detrimental effect of corners on the memory of picture pairs (Garsoffky and Schwan, 2024). In other words, pairs were better remembered if both pictures forming a pair were presented on one wall instead of across two walls. Further, pairs were better remembered if the distance between the two pictures was smaller instead of larger, a result which also replicated the findings of Garsoffky and Schwan (2024). However, there was no influence of lighting on this effect: participants always memorized picture pairs significantly better if both pictures were arranged on the same wall instead of on two adjacent walls, but, in contrast to our expectations, lighting did not influence this effect.

## 4 General discussion

Taken together, these findings show a stable effect of basic architectural features on cognitive processing: the pairs of pictures were better memorized if they were presented on one wall instead of being arranged across two adjacent walls, while there was a detrimental effect of corners on the memory of the picture pairs. This effect was found in three experiments presenting three-dimensional virtual rooms on two-dimensional screens (Garsoffky and Schwan, 2024) as well as in another two experiments using highly immersive HMD glasses (see Garsoffky et al., 2025 as well as the lighting study reported here).

Further, the studies by Garsoffky and Schwan (2024) as well as the experiment reported here, showed a significant effect of the spatial distance between the two pictures forming a pair: if this spatial distance was smaller, the pair was remembered better than if the distance was greater. This observation fits to findings on spatial clustering in memory: For example, Miller et al. (2013) in their first experiment asked participants to learn, among others, lists of well-known landmarks from all over the world, and afterward participants had to recall these landmarks in any order they wanted to. Findings showed, that landmarks that were spatially nearer to each other in the real world also had a greater probability to be remembered successively in the free recall task. This spatial clustering effect was also found for the recall of buildings and objects in a virtual town in the second experiment of Miller et al. (2013). Participants moved through the buildings of the town and delivered objects. When asked to recall the buildings, the probability was high that participants remembered buildings successively if these buildings were spatially close to each other. But, even more important, also memory for the objects showed the spatial clustering effect, i.e., participants successively remembered those objects with higher probability if they were initially learned in spatially near locations in the virtual town than in spatially more distant locations. These findings from Miller et al. (2013) fit to the effect of distance in the experiment reported in this paper: two pictures were memorized as a pair with higher probability, if they were spatially closer to each other instead of being more distant. But of course, the study reported here differs from Miller et al. (2013) in an important point, because in the tasks Miller et al. (2013) used, participants did not explicitly have to learn the spatial arrangement of the landmarks or the objects, whereas in our study, it was an explicit demand of the task to memorize where on the walls the picture pairs were.

The expected influence of lighting on pair memory was not found, i.e., there was no significant 2-way interaction between “lighting” and “wall arrangement”: based on the beam metaphor of visual attention (Eriksen and Yeh, 1985; Posner, 1980) it was expected, that if the corners of the virtual rooms were lit brighter than the middle of the walls, this could foster participants to orient their attention more toward the corners and therefore to memorize picture pairs across corners better or at least equally well than picture pairs arranged within one wall. This was not the case and there can be several reasons for that. First, maybe the lighting intensity was too low, and future experiments should vary the intensity of the beam. In contrast, the light may have even been too bright, thereby making it harder to process the pictures in the lighting beam (although this, at least subjectively, seems not likely). Second, maybe participants were irritated by the uneven lighting and tried to overcome it. So, to rule out this possibility, the instructions in future studies could explicitly emphasize that lighting is a useful hint and that participants should use the beam to orient their attention.

As a side note it should be stated clearly, the experiment presented here had a three-factorial design with the factors “wall arrangement,” “distance” and “lighting.” The calculation of the sample size was done concentrating on the main hypothesis of the study, i.e., the interaction effect of “wall arrangement” and “lighting.” There was no research question concerning an interaction of “distance” with the two other factors. The factor “distance” was only realized in the experiment, to test if former findings of a main effect of distance could be replicated, and to have some variation in the arrangement of the picture stimuli on the walls of the virtual room. Therefore, the necessary N was calculated for the two-way interaction and not for the 3-way interaction. Hence, the 3-way interaction is probably underpowered and, therefore, there is no significant effect. This does, of course not imply that there is no effect. It could be that the effect is too small to be detected with our data.

At first sight, the effect of corners might be attributed to perceptual mechanisms based on principles of Gestalt Psychology. However, these principles typically assume that the whole pictorial configuration is presented within a static field of view. In contrast, the pictorial configurations of the studies presented here required participants to move their field of view back and forth within the walls of a room by turning their body or head or by pressing the arrow-keys. Therefore, perceptual and cognitive processing of the pictorial configuration unfolded sequentially in time. As a result, room corners should not be seen as simply a static architectural element but can also be considered to induce structure in the event of experiencing the surrounding information, thereby increasing the difficulty of relating pictorial elements across adjacent walls.

This role of space was also found by Horner et al. (2016) using doors separating rooms as spatial changes. In a VR, their participants moved sequentially through several rooms by walking through doorways, and had to remember objects presented on tables in these rooms. In each room, two objects were presented lying on two tables close to the doors. Afterward participants had to decide if they had seen a specific object (initial test object in this trial) in the rooms or not. One main finding was, that when asked for a second object, objects were remembered better, if they were in the same room instead of being in the room before or after the room of the initial test object. This was the case although participants could not remember the specific rooms (they differed by color) where they initially had seen the objects. Horner et al. (2016) conclude, that the architectural feature “doorway” acted as a spatial boundary and that participants actualize their mental model when transversing such a spatial boundary. This finding can also be taken into account when looking at the effect of corners in the presented study: it is possible, that the participants always, when orienting their field of view to the next wall, updated their mental model thereby actualizing the arrangement of pictures wall by wall, and this would lead to the effect, that pairs are memorized better if arranged on one wall instead of on two adjacent walls. In other words, corners could have acted as spatial boundaries and lead to an update of the representation whenever they were crossed.

This updating of mental models at boundaries defined by changes is well known from event cognition and the event segmentation theory (Zacks and Swallow, 2007). In this model it is assumed that people continuously watch the stream of events and whenever an event inherent boundary appears (i.e., a salient change in the event), the event model is updated. Our findings on the corner effect could be interpreted tentatively in that way, implying that the process of looking around in a room is an event, and that significant changes in the environment occur, when the field of view pans to another wall, thereby inducing the impression of a boundary and leading to an update of the mental model. Taken together, room corners may act as spatial boundaries in the sense of Horner et al. (2016) and as event boundaries in the sense of Zacks and Swallow (2007) leading to a wall wise update of the picture arrangements.

Of course, for designers of physical as well as virtual learning environments, it is of great interest to understand the functioning of this effect and to see whether the influence of corners on related visual information can be overcome. Our initial empirical findings suggest that this is possible, although additional interventions may be required. The explorative findings that participants more often orient their central field of view toward the middle of the walls and not toward the corners suggest a connection between memory and viewing behavior during learning. What is more, if the viewing behavior of participants was restricted so that they were forced to look toward the corners, the detrimental effect of corners on the memory of the pairs vanished [experiment 3 in Garsoffky and Schwan (2024)]. But of course, to restrict viewers from freely choosing their viewpoint seems quite counterproductive in three-dimensional learning environments, because it is one of the major characteristics of these (mostly informal) learning settings that visitors are free to determine their own learning path and speed, thereby choosing which information they look at and in which order they do so.

Another way to overcome the corner effect on memory is based on the assumption that humans tend to group visual information together if they perceive this information as sharing a common region (Palmer, 1992). McNamara (1986) also observed that stimuli act as stronger primes for other stimuli if both stimuli fall in the same spatial region instead of being in different regions of a layout, thereby supporting the assumption that visitors develop partially hierarchical spatial mental models when moving through a three-dimensional environment. For this reason, if the goal is that a learner relates pieces of information to each other, the information can either be arranged in the same common region (e.g., on the same wall of a room) or, if this is not possible, be grouped in other ways, e.g., based on the principles postulated by Gestalt psychology (Wertheimer, 1938), which could be used to force viewers to perceive these units of information to belong to the same group. One of these principles, namely spatial proximity vs. distance, was used by Garsoffky et al. (2025); the arrangement of picture pairs in a room was not only structured by corners between the walls but in one condition some pictures were also placed closer together while others were placed at a greater spatial distance to each other. This was done by introducing a larger spatial distance between the pictures on the same wall, thereby introducing an alternative grouping possibility. Participants could either still group pictures in a wall-by-wall manner or they could group them by spatial proximity, which resulted in them perceiving pictures to the left and to the right of each corner as belonging to the same group. Our findings showed that, if there was an alternative grouping possibility which ran counter to the pictures’ separation by corners, the detrimental effect of corners on the memory of picture pairs diminished. This intervention on the arrangement of stimuli offers another opportunity for the design of informal learning settings, one which is likely to be less disturbing than restricting the learners in where to orient their central field of view, i.e., restricting their free looking behavior.

Nevertheless, in some learning settings such as (for example) historical places, even the free arrangement of pieces of information in the room may not be possible for designers. A more interesting way to compel visitors to not only perceive information in a wall-by-wall manner could be to guide their visual attention toward the corners. Based on the metaphor of the visual attentional beam (Eriksen and Yeh, 1985; Posner, 1980), in one condition of the study described here a spotlight was used to brighten the corners. But the findings showed no influence on memorizing the picture pairs; the pairs were in fact better memorized if they were arranged within one wall and worse if they were arranged on two adjacent walls independently of the lighting. In other words, there was a corner effect on memory not only in the evenly lit rooms but also in the rooms where the corners were lit more brightly than the middle of the walls. In accordance with this, the descriptive data showed that participants did not orient their central field of view more often toward the corners, even if they were lit more brightly.

Taken together, the findings of the study on the one hand replicated important significant findings, namely the significant main effects of corners and distance on memory, from previous experiments, but the expected influence of lighting on the corner effect did not become significant. This can be taken as a first hint that a simple variation in lighting may not be enough to corrupt the corner effect, but this research question should be examined in more detail in future experiments. To further examine the influence of lighting on processing of stimuli in a 3-dimensional surrounding, future experiments could, for example, work with “darkening non-important areas” instead of “brightening important ones,” or the instructions could emphasize that lighting supports to complete the task. Although, if the lighting effect is small, a higher number of participants would be necessary.

A wide range of empirical findings suggests that our spatial surroundings can have a significant influence on learning processes (for an overview, see Choi et al., 2014). This becomes especially important for learning settings in which important information is not only permanently in front of the viewer, such as in a book or on a screen, but surrounds the learner in three-dimensional space. This is the case in many virtual learning settings, but also in physical informal learning settings such as museums or historical places. One major characteristic of these places is the self-paced learning paths which the visitors choose when exploring these spatial arrangements (Falk and Dierking, 2011). Visiting such a learning environment represents an event which unfolds in time and space, and the empirical findings described in this paper suggest that even very basic architectural features such as room corners can have an impact on the learning processes of visitors. Specifically, the room corners not only seem to influence the viewing behavior of the visitors, but, more importantly, the visitors’ memory of the presented information seems to be related to these basic architectural features. If pieces of information belong together, this is better remembered if the information is arranged within one wall and not separated by a corner. Thus, we may conclude that the event of learning information arranged in space depends on physical architectural features and results in different memory content and that these processes can be influenced by careful arrangement. Curators and designers of three-dimensional informational settings can take this into account when arranging information in space.

Of course, learning encompasses several processes and they vary between learning contents and learning settings. Surely, memory is one important and basic aspect of a learning process. The findings reported here suggest an influence of architectural features on memory. But other aspects of learning were not measured in the study. Future studies should examine, if there are also other aspects of learning, that are influenced by spatial characteristics. For example, regarding event cognition, one could imagine the chronological arrangement of pictures showing an event on the walls of a room like a picture story: If the stream of events starts at one wall, and then proceeds on the adjacent wall, do the participants perceive a breakpoint in the stream of events with higher probability between the two pictures to the left and to the right of a corner than between two pictures next to each other on the same wall? Or is it possible to emphasize the internal structure of scientific processes by arranging pictures of sub-structures on one wall (for example presenting the different phases of mitosis by ordering pictures of the specific phases wall by wall) thereby supporting learners to grasp the structure? To examine the influence of architectural features on specific learning processes is important, because important content often is not only presented in a defined space in front of the learner but surrounds the learner in a physical or virtual three-dimensional room.

## Data Availability

The datasets presented in this study can be found in online repositories. The names of the repository/repositories and accession number(s) can be found below: https://osf.io/xr7ck/files/osfstorage/68a56d55e93e216be18a8a56.

## Footnotes

1
https://osf.io/xr7ck/files/osfstorage/68a56d55e93e216be18a8a56
